# 159. Transplant Transmitted Infectious Among Recipients of Drowned Donor Organs: A Six Year DTAC Experience

**DOI:** 10.1093/ofid/ofae631.045

**Published:** 2025-01-29

**Authors:** Rachel A Miller, Dong Heun Lee, Helen S Te, Anna Hughart, Maheen Abidi, Gerald J Berry, Lara A Danziger-Isakov, Cynthia E Fisher, Dzhuliyana Handarova, Chak-Sum Ho, Riki Graves, Michelle Kittleson, Charles Marboe, Marty T Sellers, Sarah Taimur, Tanvi S Sharma, Anil J Trindade, R Patrick Wood, Lorenzo Zaffiri, Tamika Watkins, Stephanie M Pouch

**Affiliations:** Duke University, Durham, North Carolina; University of California San Francisco, San Francisco, CA; Center for Liver Diseases, University of Chicago Medicine, Chicago, Illinois; Mayo Clinic, Rochester, Minnesota; University of Colorado Anschutz Medical Campus, Denver, CO; Department of Pathology, Stanford University School of Medicine, Stanford, California; Cincinnati Children's Hospital, Cincinnati, Ohio; University of Washington, Seattle, Washington; United Network for Organ Sharing, Richmond, VA; Gift of Hope Organ and Tissue Donor Network, Itasca, Illinois; Houston Methodist Hospital, Houston, Texas; Department of Cardiology, Smidt Heart Institute, Cedars-Sinai Medical Center, Los Angeles, California; NY Presbyterian Hospital/Columbia Univ Medical Center, Richmond, VA; DCI Donor Services, Inc., Nashville, Tennessee; Icahn School of Medicine at Mount Sinai, New York, NY; Boston Children's Hospital, Boston, MA; Division of Allergy, Pulmonary and Critical Care Medicine, Vanderbilt University Medical Center, Nashville, Tennessee; LifeGift Organ Donation Center, Richmond, VA; Division of Pulmonary Medicine, Cedars-Sinai Medical Center, Los Angeles, California; United Network for Organ Sharing, Richmond, VA; Emory University School of Medicine, Atlanta, GA

## Abstract

**Background:**

Characterization of transplant transmitted infections associated with drowned organ donors and the recipients is not well delineated, posing challenges for optimal management and prophylaxis strategies. Broader organ sharing may pose additional considerations due to pathogen variation.

**Methods:**

We reviewed 22 potential donor disease transmission events (PDDTEs) from drowned donors reported to the Organ Procurement and Transplantation Network (OPTN) ad hoc Disease Transmission Advisory Committee (DTAC) from 2017-2022 to estimate the incidence of PDDTEs among all US drowned organ donors and identify the most common pathogens in this setting. We also characterized donor/recipient epidemiology, infection timing and risk factors, and recipient survival.

**Results:**

From 2017-2022, drowning was the mechanism of death in 777/74,232 (1.0%) of all US organ donors, from whom 2500 organs were transplanted. Among the 22 PDDTEs, there were 70 organ recipients. The median donor age was 17.5 years (range 1-70 years) and lung recipients accounted for 5/70 organ recipients. All PDDTE reports were submitted due to recipient disease, and index cases occurred 2-19 days post-transplant. 7/22 (7/777 drowned donors, 0.9%) PDDTEs were associated with pathogens consistent with plausible waterborne transmission, of which 4 were due to invasive molds (Zygomycetes and *Scedosporium*) and 3 were due to bacteria (*Pseudomonas* and *Legionella*). Of the 21 organ recipients from these 7 donors, 8 had proven or probable infections. 6/8 recipients had severe infection with 3 deaths, all from invasive mold infections. There were no identifiable infection risk factors related to drowning water source, donor immersion time, or geographic region. However, donor/recipient regional discordance occurred in 5/21 recipients, demonstrating the trend in broader organ sharing.

**Conclusion:**

Though rare, organ recipients are at risk for transplant transmitted infection from drowned donors, particularly invasive mold infections. All infections occurred within the first month after transplant with high associated mortality. Though optimal management and prophylaxis strategies in these scenarios remain uncertain, post-transplant vigilance for early infection is paramount.

**Disclosures:**

**Helen S. Te, MD**, CVS Caremark: Advisor/Consultant **Lara A. Danziger-Isakov, MD, MPH**, Aicuris: clinical research contract, paid to institutio|Ansun BioPharma: clinical research contract, paid to institution|Astellas: Advisor/Consultant|Astellas: clinical research contract, paid to institutio|Merck: clinical research contract, paid to institutio|Pfizer: Grant/Research Support|Takeda: clinical research contract, paid to institutio **Anil J. Trindade, MD**, CareDx, Inc.: Advisor/Consultant|CareDx, Inc.: Grant/Research Support|TFF Pharmaceuticals: Advisor/Consultant|Veloxis Pharmaceuticals, Inc.: Grant/Research Support

